# The Role of the Xylem in Oxytetracycline Translocation within Citrus Trees

**DOI:** 10.3390/antibiotics9100691

**Published:** 2020-10-13

**Authors:** Faraj Hijaz, Yasser Nehela, Fuad Al-Rimawi, Christopher I. Vincent, Nabil Killiny

**Affiliations:** 1 Department of Plant Pathology, Citrus Research and Education Center, IFAS, University of Florida, 700 Experiment Station Road, Lake Alfred, FL 33850, USA; fhijaz@ufl.edu (F.H.); yasser.nehela@ufl.edu (Y.N.); falrimawi@staff.alquds.edu (F.A.-R.); civince@ufl.edu (C.I.V.); 2 Department of Agricultural Botany, Faculty of Agriculture, Tanta University, Tanta 31511, Egypt; 3 Chemistry Department, Faculty of Science and Technology, Al-Quds University, Jerusalem 90612, Palestine

**Keywords:** oxytetracycline, antibiotic, translocation, citrus, xylem

## Abstract

Antibiotics have been successfully used to control plant diseases for more than fifty years. Recently, oxytetracycline and streptomycin have been approved for the treatment of Huanglongbing, which is threatening the citrus industry in many regions. Because the efficiency of antibiotics in planta is highly affected by their movement and distribution, understanding the mechanism of antibiotics’ uptake and distribution could lead to a better control of plant pathogens. Herein, we investigated the movement of oxytetracycline within citrus plants. Oxytetracycline was applied by root drenching to both girdled and non-girdled citrus seedlings. In addition, oxytetracycline was applied by trunk injection to girdled and non-girdled citrus trees. After the exposure time (24 h), citrus seedlings were dissected and the levels of oxytetracycline in the different tissues were measured using an oxytetracycline ELISA kit. Upon root application (laboratory experiment), oxytetracycline was detected in the inner part of the stem (xylem-associated tissue), cortex (phloem-associated tissue), and leaves above and below the girdled area. Likewise, oxytetracycline was also detected in leaves of trunk-injected field trees (girdled and non-girdled) three days post treatment. Interestingly, cortex girdling did not affect the distribution and translocation of oxytetracycline, indicating that the xylem is the main path for oxytetracycline translocation. Taken together, our results indicate that oxytetracycline translocation mainly occurs via xylem vessels, and that movement into the phloem occurs subsequent to xylem translocation. Our findings also clearly demonstrated that upon trunk injection, only trace levels of oxytetracycline reached the roots, minimizing its therapeutic value there. Thus, our recommendation is to time tree injections to coincide with the flushing periods when the bacteria are moving into new shoots to maximize the efficiency of oxytetracycline.

## 1. Introduction

Citrus greening disease, also known as Huanglongbing (HLB), is currently considered to be the most destructive disease of citrus, causing massive production losses in many countries [1]. In Florida, HLB is caused by a phloem-restricted bacterium, “*Candidatus* Liberibacter asiaticus”, and is vectored by the Asian citrus psyllids, *Diaphorina citri* Kuwayama [2]. To date, there is no known cure for HLB, and control of this disease mainly relies on the control of *D. citri* using a wide range of insecticides. Several other control methods, including thermotherapy, enhanced nutritional programs, and removal of infected trees, were proposed for the management of the HLB disease. However, despite the use of these practices, HLB disease is still considered as the major threat to the citrus industry in several countries.

Antibiotics have been marketed and used for controlling a wide range of plant bacterial pathogens for more than 60 years. For example, oxytetracycline and streptomycin were used for the control of several plant diseases since the 1950s [3]. Oxytetracycline has been approved for the control of the bacterial spot disease in peaches, caused by *Xanthomonas arboricola*, and the fire blight in peach and nectarine, caused by *Erwinia amylovora* [4]. Oxytetracycline has also been used to control *Pseudomonas* spp. and *Xanthomonas* spp. in vegetables, as well as phytoplasmas that cause lethal yellow diseases in coconut palm trees and elm [4].

The use of antibiotics to control the HLB disease was started in 1970s, after it was suggested that the disease was caused by a bacterial pathogen [5]. Several studies showed that trunk injection of tetracycline reduced the HLB symptoms [6,7,8,9]. Earlier studies also showed that trunk injection was more efficient than foliar spray [10]. Trunk injection of 0.25–1.5 g/plant of achromycin resulted in the recovery of HLB-infected sweet orange seedlings [10]. Antibiotic treatments to mitigate the HLB disease have been reported in many countries, including South Africa, China, Reunion Island, and the Philippines, before they were discontinued in the beginning of the 1980s [11]. The use of antibiotics for the control of plant disease has been challenged by several factors, including inefficacy at low doses, development of phytotoxicity at higher doses, high cost of application, and development of antibiotic resistance in plant-pathogenic bacteria [12]. In addition, the improper application of antibiotics by foliar spray could influence the microbiome in the field and enhance the resistance of animal- and human-pathogenic bacteria to antibiotics [12].

However, because of the substantial losses in the citrus industry in recent years, antibiotic treatments were again recommended for the control of HLB. A recent study showed that ampicillin, penicillin, cefalexin, carbenicillin, sulfadimethoxine, and rifampicin were effective against *“Ca.* Liberibacter asiaticus” [11]. Penicillin and streptomycin were also effective in reducing the *“Ca.* Liberibacter asiaticus” titer and decreasing HLB symptoms [13]. In addition, trunk injection of penicillin G significantly decreased the “*Ca.* Liberibacter asiaticus” titer in Ray Ruby grapefruit seedlings and trees [14]. Trunk injection of oxytetracycline hydrochloride, streptomycin, and penicillin also resulted in excellent control of HLB [15].

Many factors can affect the uptake of agrochemical compounds in trees, including humidity, soil moisture, temperature, and physiological activities of the tree (time of application) [16]. In addition, the uptake of injected compounds is highly affected by the size and the type of the vascular system (xylem) [17]. The translocation of xenobiotics in plants can occur in six different ways: phloem, xylem, local, ambimobile, systemic, and translaminar [18]. Phloem movement follows the source to sink direction, can be upward (*acropetal*) or downward (*basipetal*), and is characterized by the accumulation of the agrochemical compound in shoot and root tips [19]. The xylem translocation follows the transpiration stream; it occurs upward from root or stem to leaf [20]. Ambimobile movement occurs via both the phloem and the xylem [21]. Local movement is characterized by limited movement of the applied compound [18]. The systemic movement of agrochemicals results in a complete uniform distribution of the applied compound in the plant. Finally, translaminar movement is the movement of the agrochemical compounds from one face of a leaf to the opposite face [22].

The effectiveness of agrochemicals depends on their ability to reach their target sites [18]. For instance, it is important that herbicides reach the meristem tissues to kill weeds, and insecticides should reach the phloem and xylem tissues [18]. It is believed that the xylem movement is the most effective mode of translocation of insecticides because it moves the most volume and results in a broad distribution throughout the foliage, which is attacked by chewing and sucking insects [18]. On the other hand, phloem mobility is better than xylem mobility for herbicides because it results in the accumulation of herbicides in the meristem [18].

In the same manner, the effectiveness of the antimicrobial compounds in planta also depends on their ability to reach the site of action. For example, spiroplasmas, a group of phloem-restricted plant pathogenic bacteria are sensitive to several antibiotics. However, only oxytetracycline is effective against these bacteria in planta, suggesting that it is well translocated in planta and can reach the phloem at high concentrations [23]. Trunk injection of 10–30 g of oxytetracycline showed high antibiotic activity in twigs and leaves of sweet orange trees, but the antibiotic activity was absent in the small roots [24]. In a similar study, trunk injection of oxytetracycline resulted in high antibiotic activity in the canopy but the roots showed lower or no activity [25]. On the other hand, drench treatment resulted in high antibiotic activity in the roots [25]. In the same manner, trunk injection of 6 g of oxytetracycline into coconut trees resulted in a high concentration (20 µg g^−1^ fresh weight) in the leaves, but a low level (1 µg g^−1^ fresh weight) was detected in the roots [26]. 

Oxytetracycline and streptomycin were approved for the treatment of “*Ca.* Liberibacter asiaticus”-infected trees in Florida since 2016 [27]. Thus, understanding the mechanism of their uptake and distribution in citrus is essential for a better control of the HLB disease. In our previous study, we were able to detect oxytetracycline and streptomycin in the phloem, xylem, and leaves of citrus seedlings upon root drench and stem delivery [28]. High levels of these antibiotics were detected in the canopy after stem delivery, whereas root drench resulted in high levels of these antibiotics in roots [28]. Oxytetracycline and streptomycin were detected in plant tissues thirty-five days after root drench, indicating that these antibiotics were relatively stable in citrus [28].

Xylem translocation follows the transpiration stream and occurs upward, from root or stem to leaf. Here, we hypothesize that oxytetracycline is translocated primarily via the xylem. To test this hypothesis and to determine the path (xylem vs. phloem) of oxytetracycline translocation within citrus plants, we used girdled and non-girdled plants. Girdling was used to prevent any possible transport of oxytetracycline via the phloem.

## 2. Results

### 2.1. Standard Curve

**Figure 1:**
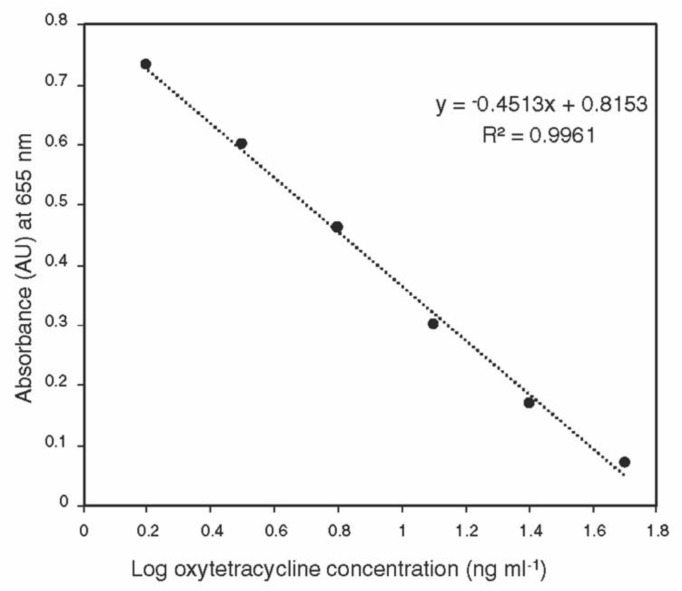
Calibration curve of oxytetracycline generated using ACCEL ELISA kit over the detection range (1.5–50 ng mL^−1^).

The standard curve generated using the ELISA kit was linear over the detection range (1.5–50 ng ml^−1^) (Figure 1). No oxytetracycline was detected in control tissues, indicating that the sample matrix does not interfere with the ELISA assay.

### 2.2. Greenhouse Experiment

**Figure 2:**
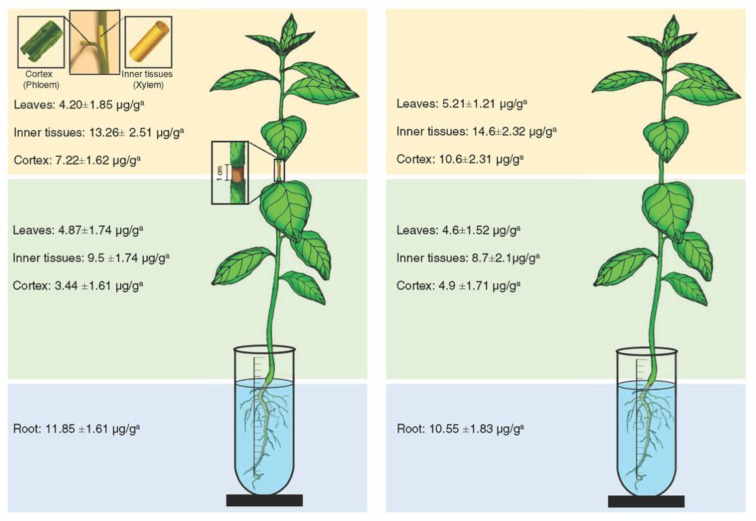
Level of oxytetracycline in different citrus plant tissues after root drench of girdled (**left**) and non-girdled (**right**) seedlings in oxytetracycline solution (200 µg mL^−1^) for 24 h. For these analyses, the stem cortex was dissected into the outer cortex tissue (representing the phloem) and the inner cortex (representing the xylem). Data are the means ± SD of five biological replicates. Means in the same line with different superscript letters are significantly different (*p*-value < 0.05).

Oxytetracycline was detected in the inner tissues (xylem), cortex (phloem), and leaves below and above the girdled area (Figure 2). The highest level of oxytetracycline was detected in the roots (11.85 ± 1.61 μ/g) (Figure 2). In the same manner, oxytetracycline was detected in the xylem, phloem, and leaves of the lower and upper part of non-girdled plants (Figure 2). The highest level of oxytetracycline in non-girdled plants was also detected in the roots (10.55 ± 1.83 μ/g) (Figure 2). No significant differences in the levels of oxytetracycline were found between girdled and non-girdled seedlings (Figure 2). In general, the level of oxytetracycline in the xylem tissues was relatively higher than that in the phloem tissues in both girdled and non-girdled plants (Figure 2).

### 2.3. Field Experiment

**Figure 3:**
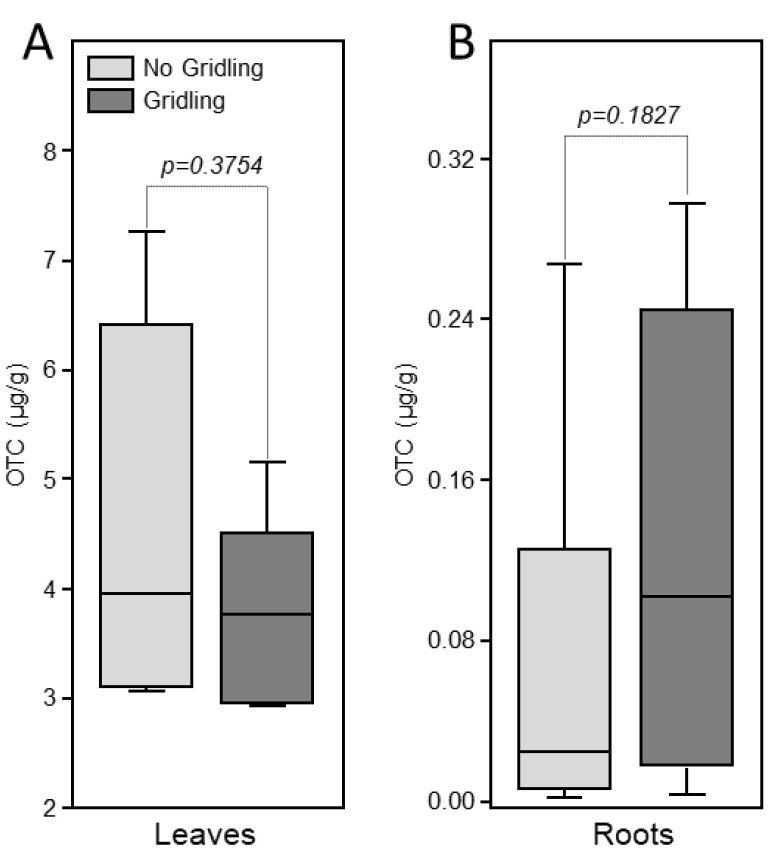
Box-plot of the levels of oxytetracycline in leaves (**A**) and roots (**B**) of citrus trees 72 h after trunk injection of 20 mL of Fireline solution (equivalent to 255 mg oxytetracycline). Data are the means ± SD of five biological replicates (field trees). Means with *p*-value > 0.05 are not significantly different using the *t*-test.

Oxytetracycline was detected in the leaves of injected trees in both girdled and non-girdled trees (Figure 3). No significant difference in the levels of oxytetracycline was observed between girdled and non-girdled trees (Figure 3A). No oxytetracycline was detected in the root of treated plants (Figure 3B).

## 3. Discussion

Like other agrochemicals, the effectiveness of the antimicrobial compounds in planta also depends on their ability to reach their site of action. Consequently, antimicrobial compounds applied to target phloem- or xylem-limited bacteria should be able to reach the phloem and xylem tissues. Oxytetracycline is slightly soluble in water (313 mg/L) with a Log Kow of −0.9, and a pka of 3.46 [29]. The physical properties of the agrochemicals such as Log Kow and pKa allow for a preliminary prediction of their transport pathways in planta [18,30]. However, several studies showed that the physical properties alone do not allow for reliable predictions in plants [18,31]. In this study, we used girdled and non-girdled plants to investigate the mobility of oxytetracycline in citrus plants. The oxytetracycline level in plant tissues was measured using an oxytetracycline ACCEL ELISA kit, which has been used and evaluated in our previous work [28]. The extraction procedure showed a good recovery of oxytetracycline from spiked tissues (85.6 ± 8.7%), and the ACCEL ELISA kit did not show any interference with the plant matrix [28].

Our current results show that the xylem was the main path of translocation for oxytetracycline in citrus plants. The girdling of the cortex tissue (phloem) of citrus seedlings and trees did not affect the translocation of oxytetracycline; oxytetracycline did not accumulate below the girdle and was detected in the cortex tissues beyond the girdle. In addition, the levels of oxytetracycline in girdled and non-girdled plants were similar. The high level of oxytetracycline in the xylem of treated seedlings indicated that oxytetracycline was xylem-mobile. Likewise, the field experiment showed that oxytetracycline was mainly translocated via the xylem, since it was detected above the girdle.

In agreement with our current results, the level of oxytetracycline in the xylem was higher than the phloem three days after treatment with oxytetracycline via root drench [28]. However, the oxytetracycline level in the phloem was higher than in the xylem seven and fourteen days after treatment, indicating that oxytetracycline was initially taken up by the xylem and then moved to the phloem [28]. The presence of oxytetracycline at high levels in the cortex above the girdle indicated that oxytetracycline in the xylem was redistributed into the phloem beyond the girdle. In agreement with our results, a bidirectional exchange between the xylem and the phloem was reported for many compounds including amino acids [32]. Our current and previous results also demonstrate that oxytetracycline is translocated to the phloem, where the “*Ca.* L. asiaticus” bacteria (a phloem-restricted pathogen) usually reside [28]. Thus, it can reach the pathogen and its associated microbiota in citrus within the phloem tissue.

Only a trace amount of oxytetracycline was detected in the roots after trunk injection, indicating that the downward movement to the root could be limited because it is against capillary action and root pressure. In agreement with these results, low levels of oxytetracycline were detected in the root after stem delivery, whereas high levels of oxytetracycline were detected in the root upon root drench [28]. Previous studies also showed that trunk injection of oxytetracycline results in high levels of oxytetracycline in the canopy and low levels in the root [24,25,26]. The results from the previous studies indicated that the downward movement of oxytetracycline was limited upon trunk injection.

Three major flush cycles were observed in citrus groves in Florida, including one main flush cycle (spring flush) and two minor flush cycles (one in the summer and one in the fall) [33]. The population dynamics of “*Ca*. L. asiaticus” is positively associated with the flushing cycles of its plant hosts [33,34,35,36,37]. Therefore, we believe that trunk injection of antibiotics during these flush cycles could be the best time to target the “*Ca*. L. asiaticus” bacteria, since they move from root to shoots during this period.

The girdling of citrus seedlings showed that the movement of oxytetracycline in citrus plants mainly occurs through the xylem and follows the transpiration stream; it occurs upward from root or stem to leaf. This conclusion was supported by three observations. First, oxytetracycline did not accumulatd below the girdle after root incubation. Second, oxytetracycline was detected beyond the girdle after root and trunk injection. Third, the level of oxytetracycline in girdled and non-girdled plants was similar.

During root incubation, oxytetracycline moves from the root to the xylem and is then redistributed into the phloem under the girdle. After passing the girdled area, oxytetracycline in the xylem can be transported to the phloem, where it can move upward or downward depending on the direction of the phloem flow (source to sink). Upon trunk injection, oxytetracycline initially moves upward in the xylem and then can be redistributed into the phloem, where it can move upward or downward depending on the direction of the phloem flow.

## 4. Materials and Methods

### 4.1. Greenhouse Study

Mexican lime (*Citrus aurantifolia*) seedlings were used in this study. The seeds were obtained from Lyn Citrus Seed, Inc. (Arvin, CA, USA) and potted in plastic cones (20 × 4 cm) containing Sungro professional growing mix (Sungro Horticulture, Agawam, MA, USA). The seedlings were maintained in a greenhouse (28 ± 1 °C, 60 ± 5% relative humidity, L16:D8 h photoperiod) at the Citrus Research and Education Center (CREC), University of Florida, Lake Alfred, Florida. The seedlings were watered twice a week. They were about five months old and about 30 ± 10 cm tall when we used them in the experiments.

**Figure 4:**
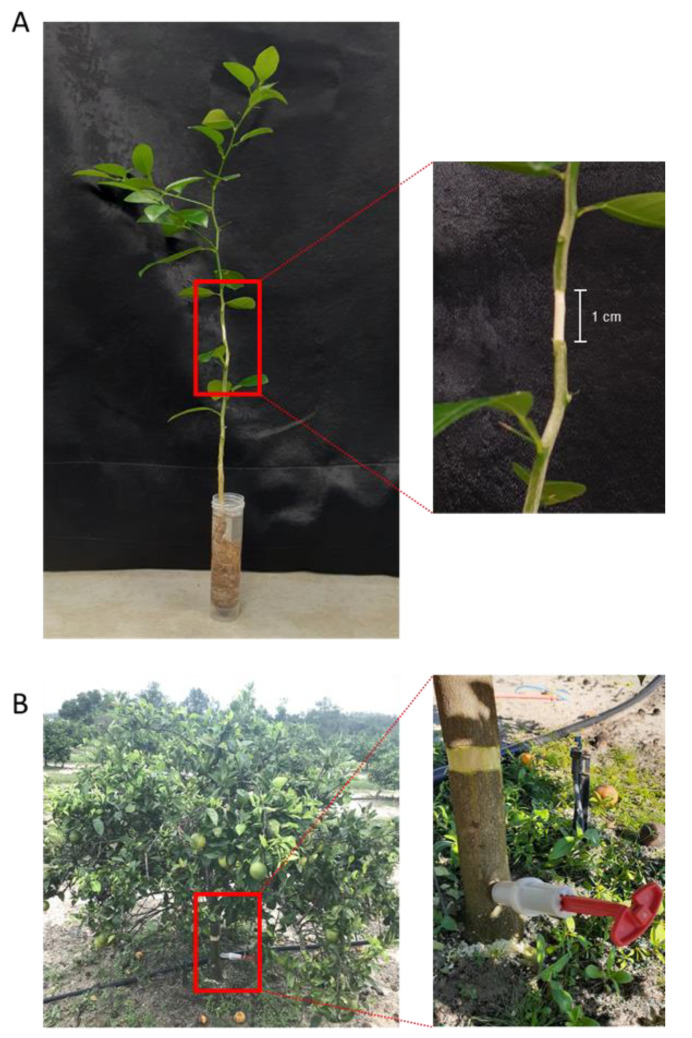
Root drench and trunk injection of oxytetracycline. Application of oxytetracycline solution (200 µg mL^−1^) to girdled (**A**) Mexican lime (*Citrus aurantifolia*) seedlings using root drenching in the laboratory. Trunk injection of oxytetracycline (255 mg per tree) into 5-year-old girdled (**B**) Hamlin trees in the field.

Oxytetracycline was obtained from Fisher Scientific (Pittsburgh, PA, USA). The oxytetracycline solution (200 µg mL^−1^) was prepared using distilled water. Five out of the ten seedlings were girdled by complete removal of a one-centimeter strip of cortex from around the entire circumference of the trunk at about 15 cm above the soil level (Figure 4A). Roots were cleaned off the soil by washing under tap water. Upon removal of the soil, the plants were immersed in 30 mL of 200 µg mL^−1^ oxytetracycline in a 50-mL plastic centrifuge tube for 24 h at room temperature (Figure 4A). Five seedlings were used for each treatment. At the end of the incubation time, seedlings were washed for 1 min with distilled water to remove any adsorbed oxytetracycline from the root surface.

After washing, the seedlings were dissected into four main parts: root, stem cortex (phloem-associated tissue), inner stem (xylem-associated tissue), and leaves. The stem of the girdled plants was further divided into two parts: upper (above the girdle) and lower (below the girdle). Likewise, the stem of non-girdled plants was also divided into two equal parts (upper and lower). The cortex (phloem-associated tissue) and the inner stem (xylem-associated tissue) were separated from each other using a sharp blade, washed with distilled water, and analyzed separately. All leaves above the girdle of each plant were collected and pooled together. All leaves below the girdle of each plant were collected and pooled together. All leaves from the upper part of each non-girdled plant were collected and pooled together (upper leaves). In the same manner, all leaves from the lower part of each plant were collected and pooled together (lower leaves). Each plant was considered as one biological replicate, and seven samples were analyzed from each plant (root, lower leaves, upper leaves, lower xylem, upper xylem, lower phloem, and upper phloem). Tissues from non-treated plants were used as a negative control.

### 4.2. Field Study

Five-year-old trees of “Hamlin” sweet orange [*Citrus sinensis* (L.) Osbeck] on Swingle citrumelo rootstocks were used for this study. The study occurred in one row of a plantation at the N40 grove located at the Citrus Research and Education Center, University of Florida, Lake Alfred, FL (28.129 latitude and −81.7178 longitude). These trees were infected with *C*Las and showed various HLB symptoms. The trees were exposed to a sandy soil with a drip irrigation system. The volume of irrigation water was 0.5 gal (1.89 L) per h for 1 h daily, from 5:00 a.m. to 6:00 a.m.

The experiment was implemented in a complete randomized design, and the trees (five for each treatment) were assigned randomly to each treatment. The selected trees were flagged and labeled using colored plastic tape. Before treatment, nine leaves were collected from each tree from three different locations (3 from the top, 3 from the middle, and 3 from the bottom).

Five out of the ten trees were girdled by complete removal of a two-centimeter strip of cortex from around the entire circumference of the trunk at about 20 cm above the soil level (Figure 4B), and five trees were left without girdling. The injection solution was prepared by dissolving 15.0 g (equivalent to 2.55 g of pure oxytetracycline) of Fireline 17 WP (Agro Source, Tequesta, FL, USA) in 200 mL of water. Before injection, a hole (45-degree angle, 40 mm long, and 4.78 mm diameter) was made in the trunk of each tree at about 10 cm above the soil level using an electrical drilling machine (Figure 4B).

A 20-mL aliquot of the Fireline solution (equivalent to 255 mg oxytetracycline) was injected in the trunk of selected trees using a 20-mL Chemjet manual injector (Healthy Tree PHC, Inc., Norfolk, VA, USA) according to the manufacturer’s instruction (Figure 4B). The injector was left until all the solution was taken by the tree (about 1 h). Three days after application, 9 leaves of each tree were collected from three different places (3 from the top, 3 from the middle, and 3 from the bottom). At the same time, three root samples were taken from three different locations around each tree (about 30 cm from the trunk and 10 cm under the soil).

### 4.3. Extraction and Analysis of Oxytetracycline

The whole plant tissues for each part from each tree were grounded using liquid nitrogen in a mortar and pestle, and about 100 mg of the homogenous sample was transferred into a 2-mL centrifuge tube. One ml aliquot of 0.1M HCl/0.01M EDTA solution was added to each tube, and the sample was vortexed for 1 min, followed by 15 min shaking and 15 min sonication. The samples were centrifuged at 12,000 rpm for 10 min at 20 °C. The supernatant was transferred into a new tube and kept at −20 °C until analysis. The oxytetracycline ACCEL ELISA kit was purchased from Plexense, Inc., (Davis, CA, USA). A 5-µL aliquot of the sample was diluted to 100 µL using the dilution buffer, which was provided with the kit, and the samples were analyzed according to the manufacturer’s instructions. A set of oxytetracycline standards (50, 25, 12.5, 6.25, 3.13, 1.56, and 0 ng mL^−1^) was prepared in the dilution buffer using the 1 mg mL^−1^ standard provided with the kit. The standard curve was constructed by plotting the absorbance (at 655 nm) for each standard on the linear *y*-axis against the logarithmic concentration on the *x*-axis, without including the 0 ng mL^−1^. The concentrations of oxytetracycline in diluted samples were determined using the equation of the standard curve and the original concentration was calculated by multiplying by the dilution factor.

### 4.4. Statistical Analysis

We used JMP 9.0 software (SAS, Cary, NC) to analyze the data. The *t*-test (*p* < 0.05) was used to compare the level of oxytetracycline in girdled plants with that in non-girdled plants.

## 5. Conclusions

Our results showed that the citrus xylem was the main route of transportation for oxytetracycline within trunk-injected citrus trees. This transport is initially unidirectional and assisted by transpiration-induced bulk flow upward into the canopy. As a matter of fact, the majority of the trunk-injected oxytetracycline will be moved upward to the canopy (*acropetally*) through the xylem. In the canopy, the oxytetracycline is distributed primarily to the branches and exchanged with the phloem laterally. However, any remaining distribution of oxytetracycline to the roots is secondary, with only limited basipetal movement through the phloem (source to sink). Our findings clearly demonstrated that only trace levels of oxytetracycline reached the roots, minimizing its therapeutic value there. Therefore, our recommendation is to time tree injections to coincide with the flushing periods (while the bacteria are moving to the canopy). This recommendation should help to improve tree health while minimizing the amount of oxytetracycline applied (maximizing efficiency) and reducing environmental exposure and off-target effects.
